# BMNet: A New Region-Based Metric Learning Method for Early Alzheimer’s Disease Identification With FDG-PET Images

**DOI:** 10.3389/fnins.2022.831533

**Published:** 2022-02-24

**Authors:** Wenju Cui, Caiying Yan, Zhuangzhi Yan, Yunsong Peng, Yilin Leng, Chenlu Liu, Shuangqing Chen, Xi Jiang, Jian Zheng, Xiaodong Yang

**Affiliations:** ^1^Institute of Biomedical Engineering, School of Communication and Information Engineering, Shanghai University, Shanghai, China; ^2^Medical Imaging Department, Suzhou Institute of Biomedical Engineering and Technology, Chinese Academy of Sciences, Suzhou, China; ^3^Department of Radiology, The Affiliated Suzhou Hospital of Nanjing Medical University, Suzhou, China; ^4^School of Biomedical Engineering, Division of Life Sciences and Medicine, University of Science and Technology of China, Hefei, China; ^5^School of Life Sciences and Technology, The University of Electronic Science and Technology of China, Chengdu, China

**Keywords:** early Alzheimer’s disease, mild cognitive impairment, FDG-PET images, bilinear pooling, inter-region representation, metric learning, embedding space

## Abstract

18F-fluorodeoxyglucose (FDG)-positron emission tomography (PET) reveals altered brain metabolism in individuals with mild cognitive impairment (MCI) and Alzheimer’s disease (AD). Some biomarkers derived from FDG-PET by computer-aided-diagnosis (CAD) technologies have been proved that they can accurately diagnosis normal control (NC), MCI, and AD. However, existing FDG-PET-based researches are still insufficient for the identification of early MCI (EMCI) and late MCI (LMCI). Compared with methods based other modalities, current methods with FDG-PET are also inadequate in using the inter-region-based features for the diagnosis of early AD. Moreover, considering the variability in different individuals, some hard samples which are very similar with both two classes limit the classification performance. To tackle these problems, in this paper, we propose a novel bilinear pooling and metric learning network (BMNet), which can extract the inter-region representation features and distinguish hard samples by constructing the embedding space. To validate the proposed method, we collect 898 FDG-PET images from Alzheimer’s disease neuroimaging initiative (ADNI) including 263 normal control (NC) patients, 290 EMCI patients, 147 LMCI patients, and 198 AD patients. Following the common preprocessing steps, 90 features are extracted from each FDG-PET image according to the automatic anatomical landmark (AAL) template and then sent into the proposed network. Extensive fivefold cross-validation experiments are performed for multiple two-class classifications. Experiments show that most metrics are improved after adding the bilinear pooling module and metric losses to the Baseline model respectively. Specifically, in the classification task between EMCI and LMCI, the specificity improves 6.38% after adding the triple metric loss, and the negative predictive value (NPV) improves 3.45% after using the bilinear pooling module. In addition, the accuracy of classification between EMCI and LMCI achieves 79.64% using imbalanced FDG-PET images, which illustrates that the proposed method yields a state-of-the-art result of the classification accuracy between EMCI and LMCI based on PET images.

## Introduction

Alzheimer’s disease (AD), a brain degenerative disorder, is harming the health of thousands of old people now, and its rate of prevalence is expected to increase rapidly in the coming decades (Wang et al., 2013; Alzheimer’s Association, 2018, 2019). Mild cognitive impairment (MCI) is considered to be a preclinical precursor of AD, but it is difficult to predict whether it will convert to AD or not (Gauthier et al., 2006; Dubois et al., 2016; Hampel and Lista, 2016). Considering the unpredictable process of MCI, it is crucial to develop relevant methods for diagnosing the early MCI and AD.

18F-fluorodeoxyglucose (FDG)-positron emission tomography (PET) can reveal altered brain metabolism in individuals with MCI and AD (Sörensen et al., 2019; Zhou et al., 2019; Wang et al., 2020). Various recent studies have proved that biomarkers derived from FDG-PET by computer-aided-diagnosis (CAD) technologies of machine learning and deep learning can accurately diagnose NC, MCI, and AD (Pagani et al., 2017; Choi et al., 2018; Blazhenets et al., 2019). Liu et al. (2018) proposed a new classification framework for AD diagnosis with 3D PET images. They decomposed 3D images into 2D slices to learn the intra-slice and inter-slice features and achieved a promising classification performance of AUC of 83.9% for MCI vs. NC classification. Zhou et al. (2021) developed a new deep belief network model for AD diagnosis based on sparse-response theory, which identified a better classification result than that of other models. To solve the multimodal data missing problem, Dong et al. (2021) proposed a high-order Laplacian regularized low-rank representation method for the classification tasks of NC, MCI, and AD. Pan et al. (2021) developed a disease-image-specific deep learning (DSDL) framework which can achieve neuroimage synthesis and disease diagnosis simultaneously using incomplete multi-modality neuroimages.

Many studies have achieved good performance on the classification of NC, MCI, and AD based on FDG-PET images. However, when it comes to the more refined task like classification of early MCI (EMCI) and late MCI (LMCI), the studies with FDG-PET images are still insufficient. Hao et al. (2020) proposed a novel multi-modal neuroimaging feature selection method with consistent metric constraint (MFCC) and obtained an accuracy (ACC) of 73.87% for the classification between EMCI and LMCI based on MRI and FDG-PET but only 64.69% when just using FDG-PET. Singh et al. (2017) proposed a multilayer neural network involving probabilistic principal component analysis for binary classification and only achieved an F1 score of 68.44%. Nozadi et al. (2018) used learned features from semantically labeled PET images to perform group classification and got an ACC of 72.5%. Forouzannezhad et al. (2018, 2020) applied a novel deep neural network and a random forest model respectively, and both models got a moderate ACC. Fang et al. (2020) introduced a supervised Gaussian discriminative component analysis (GDCA) algorithm for the effective classification of early Alzheimer’s disease with MRI and PET. Yang and Liu (2020) applied the Convolutional Architecture for Fast Feature Embedding (CAFFE) as the framework of the deep learning platform for early Alzheimer’s disease diagnosis. By comparison, based on fMRI and DTI images, Lei et al. (2020) got an ACC of 78.05% for the classification between EMCI and LMCI *via* proposing a new joint multi-task learning method by combining low-rank self-calibrated functional and structural brain networks. Song et al. (2021) constructed a new graph convolution network (GCN) and got an ACC of 79.26% based on fMRI and 82.92% based on DTI for the same classification task. With MRI images, Lian et al. (2018) developed a hierarchical fully convolutional network that can achieve an ACC of 81% for the classification between progressive MCI (pMCI) and stable MCI (sMCI).

To sum up, the refined classification performance for early AD based on FDG-PET images still has some room for improvement. One of the reasons might be that existing classification methods based on FDG-PET have not fully explored the inter-region representation among different brain regions. For example, based on fMRI, there are many methods like Pearson’s correlation and sparse representation for functional brain network (FBN) estimation (Huang J. et al., 2020). However, several studies have proved that brain metabolism connectivity has value in the diagnosis of early AD (Huang et al., 2010; Sanabria-Diaz et al., 2013; Titov et al., 2017), but few PET-based studies are using the inter-region features to improve classification performance. In addition, another reason might be that the number of PET images is generally much more than that of fMRI images in most researches. The bigger dataset might increase the variety of individuals and the probability of special samples which are hard to distinguish, thus causing complexity of the problem for classification tasks.

Considering these two limitations, we propose a novel bilinear pooling and metric learning network (BMNet) for early Alzheimer’s disease identification with FDG-PET images, especially for the classification task between EMCI and LMCI. Our main contributions are as follows: (1) We propose a shallow convolutional neural network model to achieve the classification; (2) We introduce a bilinear pooling module into the model for exploring the inter-region representation features in the whole brain; (3) We introduce the deep metric learning to help model learn the hard samples in the embedding feature space; (4) We conduct our method on the dataset collected from the publicly released ADNI database and obtain a state-of-the-art result of the classification between EMCI and LMCI based on PET images.

The rest of this paper is organized as follows. In section “Materials and Methods,” we present details of the materials and the proposed methods. Section “Results” presents the results of the experiments on the public ADNI database. Finally, we provide the discussions and conclusion of this paper in section “Discussion and Conclusion.”

## Materials and Methods

### Image Acquisition and Preprocessing

In this work, we use the data in the publicly released Alzheimer’s Disease Neuroimaging Initiative (ADNI) database (Jack et al., 2008). We collect a cohort of subjects with FDG-PET images from the ADNI databases. The ADNI cohort includes FDG-PET images from 898 subjects, including 263 NC, 290 EMCI, 147 LMCI, and 198 AD participants. Table 1 lists the demographic characteristics of subjects.

**TABLE 1:** Demographic characteristics of the subjects in the ADNI database.

Subjects	NC	EMCI	LMCI	AD
Number	263	290	147	198
Gender (M/F)	130/133	160/130	80/67	119/79
Age	75.49 ± 6.47	71.40 ± 7.33	72.16 ± 7.55	75.05 ± 7.60
MMSE	29.06 ± 1.13	28.32 ± 1.57	27.62 ± 1.84	23.20 ± 2.17

*The values are presented as mean ± standard deviation.*

*MMSE, Mini-Mental State Examination.*

We choose FDG-PET images which are in a state of rest with 30–35 min with 185 ± 18.5 MBq FDG, and details of acquisition can be obtained from the study protocols in the ADNI database. Firstly, we normalize the images based on the template of the Montreal Neurological Institute (MNI). Then, we perform the smoothing with a Gaussian filter of 8 mm fullwidth at half-maximum (FWHM) (Wang et al., 2020). Finally, to verify the effectiveness of the proposed method, we do the main experiments using two different brain atlas. Based on the automated anatomical labeling (AAL) (Ashburner and Friston, 2000) atlas, we extract features of 90 regions of interest (ROIs) from FDG-PET images with intensity normalized averagely. Similarly, based on the Schaefer et al. (2018) atlas (Schaefer et al., 2018), we extract features of 400 regions. We perform all preprocessing steps by Statistical Parametric Mapping software (SPM12) (Tzourio-Mazoyer et al., 2002) and Matlab (2020).

### Methods

#### Overview of the Proposed Network

**FIGURE 1:**
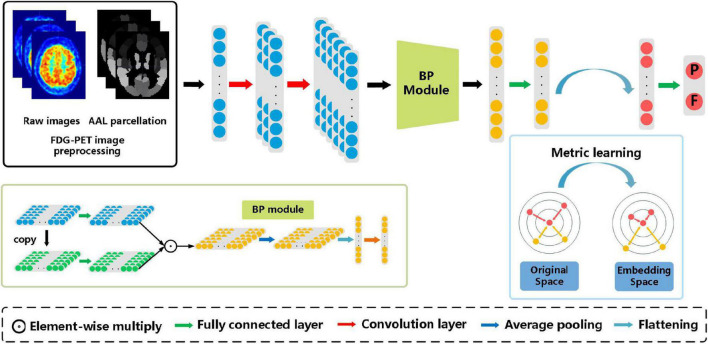
The architecture of the proposed bilinear pooling and metric learning network (BMNet) for MCI diagnosis using PET images. There are four modules in our framework (i.e., images preprocessing module, convolutional feature-extraction module, bilinear pooling module, and the metric learning module).

Figure 1 illustrates the method framework of this study. The left box is the preprocessing step of FDG-PET images, in which the left image is the raw PET image of the brain, and the right one is the AAL template. Then 90 features extracted based on the AAL template are input into the subsequent model. The model consists of two convolution layers, a bilinear pooling layer, and two fully connected layers.

After extracting the first-order features through two convolution layers, the bilinear pooling module is used to further extract the inter-region-based features. Finally, the metric learning loss is added to the classification loss to strengthen the ability to learn hard samples of the proposed model.

#### Baseline Model

We construct a shallow neural network as the Baseline model, including two convolution blocks and three fully connected layers. Each convolution block includes a convolution layer, a batch normalization layer, and a Rectified Linear Unit (ReLU) activation layer.

Given a set of nodes (regions) *R* = {r1, r2, r3}, and the features of each region is denoted as *X_i_*. Each convolution block is defined as:


(1) $$Y_{i}=\sigma \left[BN\left(f\left(X_{i}\right)\right)\right]$$

Where the *f* represents the convolution process, *BN* represents the batch normalization process, σ represents the activation process.

#### Generating Inter-Region Representation *via* Bilinear Pooling Module

In this section, we propose to use a bilinear pooling module to further generate second-order features which may represent inter-region features among whole brain regions. Bilinear pooling is an effective feature fusion method, which has been widely used in various computer vision and machine learning tasks (Lin et al., 2015; Gao et al., 2020). Bilinear pooling captures the high-order statistical information of features by matrix operations and then generates an expressive global representation (Kim et al., 2016; Li et al., 2017; Gao et al., 2020). In the research of DTI and fMRI, this method is also used to extract connectivity-based features between brain regions (Huang F. et al., 2020). In theory, by using these features, the inter-region representation among the whole brain regions in FDG-PET images could be exploited to some extent, as the functional brain network of fMRI.

In this work, we introduce a new factorized bilinear pooling method (Gao et al., 2020) to capture inter-region features by fusing homogeneous features where the input features are from the same source. This new bilinear pooling method simplifies the complexity of calculation, reduces heavy computational redundancy issues. Based on factorized bilinear coding, it is proved that bilinear features are rank-one matrices whose rank is one. The bilinear features could be extracted by factorizing dictionary atoms into low-rank matrices and Hadamard product, instead of massive matrix operations, reducing the dimension of matrices and computational burden.

The main operations of bilinear features are as follows (Kim et al., 2016; Gao et al., 2020):


(2) $$B=Y^{T}WY=Y^{T}UV^{T}Y=P^{T}\left(U^{T}Y{}^{\circ}V^{T}Y\right)$$

where *B* represents the bilinear features, and *Y* represents the input feature, *U^T^*, *V^T^* and *P^T^* are learnable parameters of the dictionary, ° represents Hadamard product.

The low-rank matrix *U* and *V* are used to approximate *W*, and the operation is simplified. Matrix *P* is used to control the length of the output. In the network, three fully connected layers are used to learn *U^T^*, *V^T^* and *P^T^*. Then, we use an average pooling layer to diminish the feature dimension and obtain the global information. Finally, the feature map is flattened to one-dimensional and a fully connected layer is used to diminish the feature dimension to facilitate subsequent learning processes.

We use this bilinear pooling method to capture inter-region representation with FDG-PET images. The homogeneous features achieve interaction of the whole brain by the bilinear pooling module, which needs complex and expensive computation before.

#### Distinguishing Hard Samples in Embedding Space by Metric Learning

In this section, we introduce the deep metric learning strategy into the classification of different stages of AD. Metric learning is widely utilized with deep neural networks in classification tasks, especially in problems affected by large intra-class sample changes (Liu et al., 2017; Sundgaard et al., 2021). Deep metric learning loss maps features to the embedded space, which is conducive to learning difficult samples and can effectively deal with the imbalance of data (Sundgaard et al., 2021). Inspired by these, we argue that deep metric learning might be suitable for our classification task. Thus, in this paper, we employ deep metric learning for the diagnosis of AD to help distinguish hard samples in the embedding space.

In deep neural networks, the loss function is a manifestation of metric learning, and there are a variety of different metric learning loss functions. In this paper, we employ two deep metric learning loss functions for automatic diagnosis of early AD, including contrastive loss and triplet loss, which are widely used in recent studies (Cheng et al., 2016; He et al., 2021; Sundgaard et al., 2021). Contrastive loss employs a pair of positive and negative samples for each training iteration. The contrastive loss function is measured by the Euclidian distance between two vectors in embedding space. The contrastive loss function is given as (Hadsell et al., 2006):


(3) $$L_{c}\left(b_{1,i},b_{2,i}\right)=\Sigma_{i=1}^{N}\left[y_{i}d_{1,2}^{2}+\left(1-y_{i}\right){\left\{max\left(0,m-d_{1,2}\right)\right\}}^{2}\right]$$

(4) $$d_{1,2}={||f_{1,i}-f_{2,i}||}_{2}^{2}$$

where *y*_*i*_ = 0 for two positive vectors and *y*_*i*_ = 1 for negative pairs, *b*_*1,i*_, *b*_*2,i*_ is the training input from two classes, *f*_1,*i*_, *f*_*2,i*_ represents the embedding vector of each training input generated by the network, *N* is the number of input samples, and *m* is the margin, usually set to 1.0.

When the input is a positive sample pair, *d*_*1,2*_ decreases gradually, and the same kind of samples will continue to form clusters in the feature space. On the contrary, when the network inputs a negative sample pair, *d*_*1,2*_ will gradually rise until it reaches the set *m*. By minimizing the loss functions, the distance between positive sample pairs can be gradually reduced and the distance between negative sample pairs can be gradually increased, to meet the needs of the classification task.

Triplet loss is a widely used measure of metric learning loss, which is the basis of a large number of metric learning methods. Unlike contrastive loss, triplet loss requires three input samples including two positive samples and a negative sample. The three samples are named as fixed sample (anchor) *b^a^*, positive sample (positive) *b^p^* and negative sample (negative) *b^n^* respectively. *b^a^* and *b^p^* form positive sample pairs, and *b^a^* and *b^n^* form negative sample pairs.

This triplet loss function simultaneously penalizes a short distance *d*_*a,n*_ between an anchor and a negative sample and a long distance *d*_*a,p*_ between an anchor and a positive sample, and is defined as (Schroff et al., 2015):


(5) $$L_{triplet}\left(b_{i}^{a},b_{i}^{p},b_{i}^{n}\right)=\Sigma_{i=1}^{N}max\left(0,m+d_{a,p}-d_{a,n}\right)$$

where $b_{i}^{a}$, $b_{i}^{p}$, $b_{i}^{n}$ is the input from two training groups, *N* represents the number of samples, and *m* is the margin, usually set to 1.0.


(6) $$d_{a,p}={||f_{i}^{a}-f_{i}^{p}||}_{2}^{2}$$

(7) $$d_{a,n}={||f_{i}^{a}-f_{i}^{n}||}_{2}^{2}$$

$f_{i}^{a}$, $f_{i}^{p}$, $f_{i}^{n}$ represents the vector of training input in embedding space.

As shown in Figure 1, the triple loss can shorten the distance between positive sample pairs, while pushing away the distance between negative sample pairs. Finally, samples with the same class form feature clusters and embedding space to improve the performance of the classification tasks.

#### Loss Functions

In addition, we use cross-entropy loss *L*_*C*_ for the classification task. Therefore, the final loss function includes a joint loss function *L*_*total*_ that contains metric loss *L*_*M*_ for the embedding space and cross-entropy loss for the classification task.


(8) $$L_{total}=\lambda L_{M}+L_{C}$$

(9) $$L_{C}=\frac{1}{N}\Sigma_{i}^{N}-\left[y_{i}log\left(p_{i}\right)+\left(1-y_{i}\right)log\left(1-p_{i}\right)\right]$$

Where *y*_*i*_ represents the label of the sample *i*, where *p*_*i*_ represents the probability that the sample *i* is projected to be a positive class, λ represents the coefficient which we define as 0.05 by experience.

#### Performance Evaluation

We adopt six commonly used evaluation metrics to evaluate the performance of the models objectively, including accuracy (ACC), sensitivity (SEN), specificity (SPE), positive predictive value (PPV), negative predictive value (PPV), F1 score (F1), area under the receiver operating characteristic curve (AUC).

#### Implementation Details

We implement the proposed network based on the public platform PyTorch 1.8 and Intel Core i5-9400 CPU with 16 GB memory. Besides, we adapt stochastic gradient descent (SGD) to optimize the model, in which momentum and weight decay are set to 0.9 and 0.001 respectively.

#### Validation Strategies and Statistic Analysis Methods

To evaluate the effectiveness of the proposed model, we conduct a fivefold cross-validation strategy in all ablation and comparative experiments based on the AAL atlas. For each experiment, we divide data into five groups, and each group maintains the same proportion of two classes. In each fold experiment, four groups are used as train groups and another group is used as the test group. The detailed classification results on the ADNI database are summarized in section “Ablation Experiments.”

In addition, we apply independent testing set strategy in the experiments based on Schaefer et al. (2018) atlas. We divide the collected dataset from the ADNI database into a training set (80%), validation set (10%), and testing set (10%). The corresponding detailed classification results are summarized in sections “Experiments on Different Atlases.”

Similarly, to evaluate the effectiveness of the proposed model, we use two methods to validate the statistical significance including the *t*-test and DeLong test. In the experiments on the AAL atlas, we use the *t*-test. In the experiments on Schaefer et al. (2018) atlas, we use the DeLong test.

## Results

### Ablation Experiments

**FIGURE 2:**
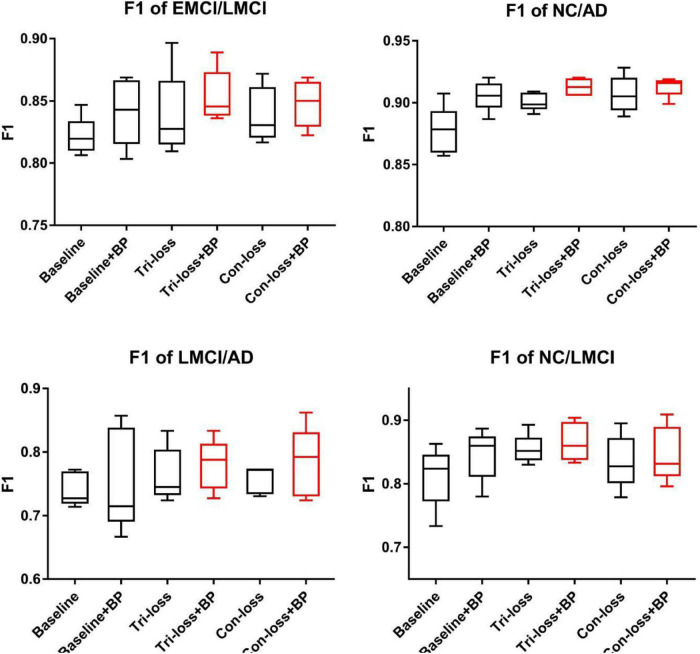
The F1 scores of experiments for EMCI vs. LMCI classification, NC vs. LMCI classification, LMCI vs. AD classification and NC vs. AD classification.

To verify the effect of the bilinear pooling module and the metric learning loss on the performance of the proposed model, we remove the bilinear pooling module and the metric learning mechanism loss from the proposed BMNet, respectively. In the first experiment (i.e., our method without a bilinear pooling module), we directly use a fully connected layer to replace the bilinear pooling module. In the second experiment (i.e., our method without metric learning losses), we just use the cross-entropy loss function. The details are as follows and the results are shown in Tables 2–5 and Figure 2.

**TABLE 2:** Results of the ablation studies of BP module and metric learning losses for EMCI vs. LMCI classification (Mean ± Standard Deviation).

Method	ACC (%)	PPV (%)	NPV (%)	SEN (%)	SPE (%)	AUC	F1 (%)	p
Baseline	75.74 ± 2.96	83.79 ± 2.32	59.84 ± 13.06	80.79 ± 4.89	64.92 ± 2.09	0.7332 ± 0.0602	82.26 ± 1.53	–
Baseline + BP	78.48 ± 3.44	86.21 ± 4.22	63.29 ± 4.18	82.24 ± 2.03	70.29 ± 7.26	0.7629 ± 0.0719	84.17 ± 2.70	0.068
Tri-loss	77.35 ± 5.28	87.93 ± 5.72	56.34 ± 19.67	80.49 ± 6.45	71.3 ± 8.60	0.7415 ± 0.0963	84.05 ± 3.41	0.342
Tri-loss + BP	**79.64 ± 3.11**	**89.31 ± 2.56**	60.55 ± 9.54	81.84 ± 3.66	**74.29 ± 4.18**	0.7589 ± 0.0633	**85.41 ± 2.13**	0.013*
Con-loss	77.81 ± 3.05	86.55 ± 3.57	59.17 ± 9.13	80.88 ± 3.37	70.53 ± 5.67	0.7387 ± 0.0801	83.94 ± 2.22	0.342
Con-loss + BP	79.40 ± 1.92	86.90 ± 6.41	**64.67 ± 10.58**	**83.22 ± 3.86**	72.43 ± 6.59	**0.7707 ± 0.0848**	85.01 ± 1.89	0.079

*The bold values represent the highest number.*

*The asterisk represents the results have the statistical significance.*

**TABLE 3:** Results of the ablation studies of BP module and metric learning losses for NC VS. AD classification (Mean ± Standard Deviation).

Method	ACC (%)	PPV (%)	NPV (%)	SEN (%)	SPE (%)	AUC	F1 (%)	p
Baseline	85.25 ± 2.50	92.01 ± 2.82	76.33 ± 6.77	83.91 ± 3.96	87.99 ± 3.52	0.9074 ± 0.0215	87.77 ± 1.96	–
Baseline + BP	88.94 ± 1.20	93.52 ± 3.48	82.79 ± 3.50	87.92 ± 1.77	90.93 ± 4.33	0.9286 ± 0.0218	90.63 ± 1.22	0.051
Tri-loss	88.29 ± 0.86	93.54 ± 0.99	81.33 ± 2.04	86.92 ± 1.44	90.47 ± 1.31	0.9279 ± 0.0127	90.35 ± 0.74	0.059
Tri-loss + BP	**89.80 ± 0.62**	93.53 ± 3.70	**84.81 ± 4.88**	**89.28 ± 2.79**	91.20 ± 4.28	0.9281 ± 0.0192	91.11 ± 0.70	0.032*
Con-loss	89.14 ± 1.75	93.53 ± 2.56	83.35 ± 2.78	88.02 ± 1.59	90.76 ± 3.54	0.9281 ± 0.0253	90.69 ± 1.48	0.088
Con-loss + BP	**89.80 ± 0.99**	**93.90 ± 2.44**	84.32 ± 3.40	88.91 ± 2.00	**91.40 ± 2.97**	**0.9334 ± 0.0141**	**91.35 ± 0.80**	0.029*

*The bold values represent the highest number.*

*The asterisk represents the results have the statistical significance.*

**TABLE 4:** Results of the ablation studies of BP module and metric learning loss for NC VS. LMCI classification (Mean ± Standard Deviation).

Method	ACC (%)	PPV (%)	NPV (%)	SEN (%)	SPE (%)	AUC	F1 (%)	p
Baseline	76.81 ± 4.27	78.86 ± 9.26	73.45 ± 10.68	84.50 ± 4.26	66.81 ± 6.33	0.7527 ± 0.0520	81.58 ± 4.79	–
Baseline + BP	80.00 ± 4.61	86.28 ± 6.49	68.71 ± 8.88	83.25 ± 3.83	74.45 ± 7.40	0.7871 ± 4.57	84.74 ± 4.03	<0.001*
Tri-loss	80.49 ± 2.86	89.33 ± 4.01	64.60 ± 5.42	81.91 ± 2.04	77.68 ± 6.17	0.7702 ± 5.77	85.46 ± 2.34	0.007*
Tri-loss + BP	**82.20 ± 4.36**	**89.36 ± 3.17**	69.42 ± 11.53	84.18 ± 5.23	**78.48 ± 5.76**	0.7985 ± 5.38	**86.69 ± 3.05**	<0.001*
Con-loss	79.03 ± 4.83	83.22 ± 7.72	71.36 ± 13.62	84.31 ± 5.15	71.28 ± 6.79	0.7841 ± 4.18	83.76 ± 4.23	0.016*
Con-loss + BP	81.46 ± 3.99	84.64 ± 6.62	**72.02 ± 9.80**	**84.96 ± 3.64**	76.05 ± 7.78	**0.8096 ± 4.22**	84.80 ± 4.35	0.001*

*The bold values represent the highest number.*

*The asterisk represents the results have the statistical significance.*

**TABLE 5:** Results of the ablation studies of BP module and metric learning loss for LMCI vs. AD classification (Mean ± Standard Deviation).

Method	ACC (%)	PPV (%)	NPV (%)	SEN (%)	SPE (%)	AUC	F1 (%)	p
Baseline	77.69 ± 2.67	74.16 ± 5.54	80.30 ± 6.73	74.57 ± 5.91	80.86 ± 2.54	0.7964 ± 0.0216	74.37 ± 2.67	–
Baseline + BP	80.06 ± 5.78	72.92 ± 11.12	**85.37 ± 4.11**	78.62 ± 5.95	81.29 ± 6.67	0.8108 ± 0.0581	75.66 ± 8.02	0.243
Tri-loss	80.60 ± 3.04	74.25 ± 9.76	85.31 ± 6.67	**79.77 ± 6.85**	82.23 ± 5.29	0.8040 ± 0.0422	76.91 ± 4.30	0.307
Tri-loss + BP	81.18 ± 2.72	**78.90 ± 7.80**	82.85 ± 4.39	77.53 ± 3.26	**84.41 ± 4.42**	0.8167 ± 0.0295	78.21 ± 3.97	0.022*
Con-loss	79.71 ± 0.97	74.87 ± 7.56	83.31 ± 5.89	77.53 ± 5.36	82.00 ± 3.37	0.8018 ± 0.0332	76.18 ± 2.19	0.327
Con-loss + BP	**81.77 ± 4.50**	77.54 ± 8.19	84.91 ± 6.27	79.64 ± 6.88	83.84 ± 4.97	**0.8297 ± 0.0346**	**78.57 ± 5.53**	0.028*

*The bold values represent the highest number.*

*The asterisk represents the results have the statistical significance.*

#### The Ablation Experiments of the Bilinear Pooling Module

Firstly, we conduct the experiments based on the Baseline model. Then we conduct the experiments of adding a bilinear pooling (BP) module to the Baseline model. According to the results, after the BP module is added, the four groups of classification experimental results have been improved to a certain extent. Specifically, in classification experiments between EMCI and LMCI, ACC increases by 2.74%, and AUC increases by 2.97%. In classification experiments between NC and AD, the results are the best, where ACC increases by 3.69% and AUC increases by 2.12%. In addition, we also conduct experiments in the classification between NC and LMCI, LMCI, and AD. The results illustrate that the BP module has a good generalization ability in the different classification tasks.

Furthermore, we conduct comparative experiments to verify the effectiveness of the BP module based on metric learning loss. For example, in the classification experiments of EMCI and LMCI, after adding the BP model to the triplet loss (Tri-loss), ACC increases by 2.29%, and AUC increases by 1.74%.

#### The Ablation Experiments of Metric Learning Losses

In this sub-section, we perform comparative experiments in terms of metric learning losses, including the triplet loss (Tri-loss) and the contrastive loss (Con-loss). We use two kinds of metric learning losses respectively, and the results illustrate that the two metric learning losses are both effective in different experiments. Specifically, in the classification experiments between EMCI and LMCI, ACC increases by 2.07% after adding the contrastive loss, which is a little higher than that of triplet loss. Similarly, in the classification experiments between NC and AD, ACC increases by 3.89%. In the classification experiments between NC and LMCI, the results of triplet loss improve more than these of contrastive loss, and ACC reaches 0.8049. On the contrary, in the classification experiment between LMCI and AD, the results of contrastive loss are better, where ACC reaches 0.8177 and AUC reaches 0.8297.

Finally, we use the *t*-test to measure the statistical significance comparing AUCs and the results are shown as *p*-value in Tables 2–5. We can see that most results of the two final models (Con-loss + BP and Tri-loss + BP) are statistically significant. In addition, we can also see that most F1 scores of the two final models are higher than these of other models in Figure 2.

### Experiments on Different Atlases

**FIGURE 3:**
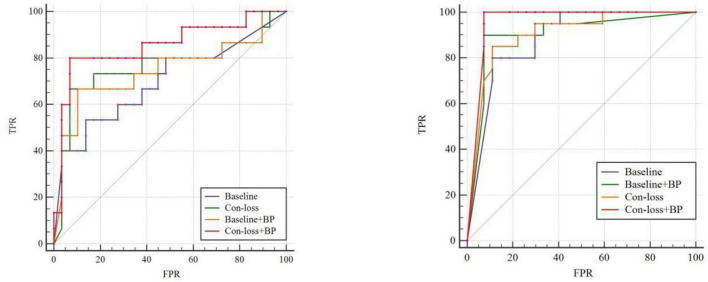
Receiver operating characteristic (ROC) curves of experiments for EMCI vs. LMCI classification and the ROC of experiments for NC vs. AD classification based on the Schaefer et al. (2018) atlas. TPR, true positive rate; FPR, false-positive rate; AUC, area under the receiver operating characteristic curve. Please see the web version for the complete colorful picture.

In this section, we evaluate the performance of our method (Con-loss + BP) based on the Schaefer et al. (2018) atlas. We conduct two groups of experiments for EMCI vs. LMCI classification and NC vs. AD classification and the results are shown in Table 6 and Figure 3. As stated earlier, we apply independent testing set strategy in these experiments and use the DeLong test to validate the statistical significance.

**TABLE 6:** Results of the main studies based on the Schaefer et al. (2018) atlas.

Class	Method	ACC	SEN	SPE	F1	AUC	*p*
EMCI-LMCI	Baseline	0.7500	0.7647	0.7000	0.8254	0.7379	0.0358*
	Con-loss	0.7727	0.7714	0.7778	0.8438	0.7609	0.1090
	Baseline + BP	0.7955	0.8125	0.7500	0.8525	0.7425	0.0990
	Con-loss + BP	**0.8409**	**0.8235**	**0.9000**	**0.8889**	**0.8529**	–
NC-AD	Baseline	0.8298	0.8519	0.8000	0.8519	0.8796	0.0395*
	Con-loss	0.8511	0.8571	0.8421	0.8727	0.9139	0.3212
	Baseline + BP	0.8511	0.8333	0.8824	0.8772	0.9259	0.3548
	Con-loss + BP	**0.8936**	**0.8929**	**0.8947**	**0.9091**	**0.9574**	–

*The bold values represent the highest number.*

*The asterisk represents the results have the statistical significance.*

The results illustrate that both BP module and contrastive loss are effective based on the Schaefer et al. (2018) atlas. In the experiments for EMCI vs. LMCI classification, ACC increases by 2.27% after adding the contrastive loss, which is a little lower than that of the BP module. Similarly, in the classification experiments between NC and AD, ACC increases by 2.13%. Finally, combining the BP module and contrastive loss, the final model (Con-loss + BP) achieves much improvement in both two classification experiments. Specifically, in the classification experiments for EMCI and LMCI, ACC, SEN, SPE, F1 and AUC achieve 84.09%, 82.35%, 90%, 88.89% and 0.8529 with an improvement of 9.09, 5.88, 20, 6.35, and 11.5% respectively, compared with Baseline model. In the NC vs. AD classification experiments, ACC, SEN, SPE, F1 and AUC increases by 6.38%, 4.1%, 9.47%, 5.72%, 7.78% and reach 89.36%, 89.29%, 89.47%, 90.91% and 0.9574.

### Comparison With Other Methods

In this section, we compare the performance of our method (Tri-loss + BP) with that of several recent representative methods. In addition, we apply the least absolute shrinkage and selection operator (LASSO) feature selection method and support vector machine (SVM) method for the contrast experiments. From Table 7, we can find that our method gets the highest performance in classification experiments between EMCI and LMCI based on FDG-PET images. Specifically, the proposed method yields big improvement than the results of Singh et al. (2017) and Nozadi et al. (2018), although the dataset in our experiments is highly unbalanced. Based on a similar dataset, the proposed method still has better performance than the methods proposed by Forouzannezhad et al. (2018, 2020). In addition, compared with the method proposed by Hao et al. (2020), our method achieves an overall huge improvement with 14.95% in ACC, 3.67% in SEN, 29.85% in SPE, and 12.89% in AUC, respectively. Compared to the results of the fusion of PET and MRI (Singh et al., 2017; Forouzannezhad et al., 2018, 2020; Nozadi et al., 2018; Fang et al., 2020; Hao et al., 2020), our method also achieves an improvement in most metrics. Besides, our method gets a comparable performance compared to the methods based on fMRI and DTI adapted by Lei et al. (2020) and by Song et al. (2021), but the subjects in our research are much more than those they use.

**TABLE 7:** Comparison of the performance of different model algorithms in experiments for EMCI vs. LMCI classification with the related works.

Method	Modality	DATA (EMCI/LMCI)	ACC	SEN	SPE	AUC	F1
SVM	PET	290/147	0.6620	0.7769	0.4653	0.6329	–
Singh et al., 2017	PET	178/158	–	0.6482	–	–	0.6844
Nozadi et al., 2018	PET	164/189	0.7250	0.7920	0.6990	0.790	–
Forouzannezhad et al., 2018	PET	296/193	0.6230	0.7820	0.4000	–	–
Forouzannezhad et al., 2020	PET	296/193	0.6280	0.6150	0.6430	–	–
Yang and Liu, 2020	PET	–	0.7219	0.7382	0.7305	–	–
Hao et al., 2020	PET	273/187	0.6469	0.7817	0.4444	0.6300	–
	PET+MRI		0.7387	0.9055	0.4952	0.7000	–
Fang et al., 2020	PET+MRI	297/196	0.8333	0.8235	0.8966	0.8947	
Lei et al., 2020	fMRI	44/38	0.7805	0.7368	0.8182	0.8571	–
	DTI		0.5366	0.5789	0.5000	0.5260	–
Song et al., 2021	fMRI	44/38	0.7926	0.8421	0.7500	0.9067	–
	DTI		**0.8292**	**0.9473**	0.7272	**0.9414**	–
Our method (Tri-loss+BP)	PET	290/147	0.7964	0.8184	0.7429	0.7589	**0.8541**
Our method (Con-loss+BP on Schaefer atlas)	PET	290/147	**0.8409**	**0.8235**	**0.9000**	**0.8889**	0.8529

*The bold values represent the highest number.*

Similarly, from Table 8, we can find that our method gets the highest performance of classification experiments between NC and AD based on PET images too. Specifically, compared with the method proposed by Hao et al. (2020) based on PET images, our method achieves an overall huge improvement with 9.74% in ACC, 3.26% in SEN, 19.26% in SPE, and 7.81% in AUC, respectively. Besides, our method gets a comparable performance compared to the methods based on other modalities (Lian et al., 2018; Lei et al., 2020; Song et al., 2021). ACC, SEN, SPE, AUC of our method based on PET images improve 10.76%, 4.69%, 16.83%, and 2.82% than those of their method based on fMRI. While SEN and AUC are slightly lower, ACC and SPE based on PET images improve 7.1% and 19.11% than those based on DTI.

**TABLE 8:** Comparison of the performance of different model algorithms in experiments for NC vs. AD classification with the related works.

Method	Modality	DATA (NC/AD)	ACC	SEN	SPE	AUC
SVM	**PET**	263/198	0.6213	0.8063	0.5547	0.8445
Hao et al., 2020	**PET**	211/160	0.8006	0.8602	0.7194	0.85
	MRI		0.8663	0.9028	0.8181	0.93
Lei et al., 2020	fMRI	44/38	0.7805	0.7368	0.8182	0.8571
	DTI		0.5366	0.5789	0.5000	0.5260
Song et al., 2021	fMRI	44/38	0.7926	0.8421	0.75	0.9067
	DTI		0.8292	0.9473	0.7272	0.9414
Lian et al., 2018	MRI	429/358	0.90	0.82	**0.97**	0.95
Dong et al., 2021	MRI+PET	440/367	**0.9305**	**0.9474**	**0.9091**	0.9732
Our method (Tri-loss+BP)	**PET**	263/198	**0.898**	0.8928	**0.912**	0.9281
Our method (Con-loss+BP on Schaefer atlas)	**PET**	263/198	0.8936	**0.8929**	0.8947	**0.9574**

*The bold values represent the highest number.*

In addition, we conduct the classification experiments between NC and LMCI, LMCI and AD, and the results compared with other methods are shown in Tables 9, 10 respectively, to further validate the effectiveness of our method.

**TABLE 9:** Comparison of the performance of different model algorithms in experiments for NC vs. LMCI classification with the related works.

Method	Modality	DATA (NC/LMCI)	ACC	SEN	SPE	AUC
SVM	**PET**	263/147	0.6415	0.7446	0.5437	0.6724
Hao et al., 2020	**PET**	273/187	0.6677	0.7545	0.5594	0.68
	MRI		0.712	0.7801	0.6332	0.76
Lei et al., 2020	Fmri	44/38	0.7805	0.7368	**0.8182**	0.8571
	DTI		0.5366	0.5789	0.5000	0.5260
Song et al., 2021	fMRI	44/38	0.7926	0.8421	0.75	0.9067
	DTI		**0.8292**	**0.9473**	0.7272	**0.9414**
Our method (Tri-loss+BP)	**PET**	263/147	0.822	0.8418	0.7848	0.7985

*The bold values represent the highest number.*

**TABLE 10:** Comparison of the performance of different model algorithms in experiments for LMCI vs. AD classification with the related works.

Method	Modality	DATA (LMCI/AD)	ACC	SEN	SPE	AUC
SVM	**PET**	147/198	0.5841	0.7834	0.5044	0.6908
Hao et al., 2020	**PET**	273/187	0.6677	0.7545	0.5594	0.68
	MRI		0.712	0.7801	0.6332	0.76
Lei et al., 2020	fMRI	44/38	0.7805	0.7368	0.8182	0.8571
	DTI		0.5366	0.5789	0.5000	0.5260
Song et al., 2021	fMRI	44/38	0.7926	0.8421	0.75	0.9067
	DTI		**0.8292**	**0.9473**	0.7272	**0.9414**
Our method (Tri-loss+BP)	**PET**	147/198	0.8118	0.7753	**0.8441**	0.8167

*The bold values represent the highest number.*

From those experiments above, we can see that our classification results between EMCI and LMCI have exceeded those of the existing methods overall based on FDG-PET images. In addition, our results are also comparable with those based on fMRI and DTI images.

## Discussion and Conclusion

### Comparison of Different Coefficients in Loss Functions

**FIGURE 4:**
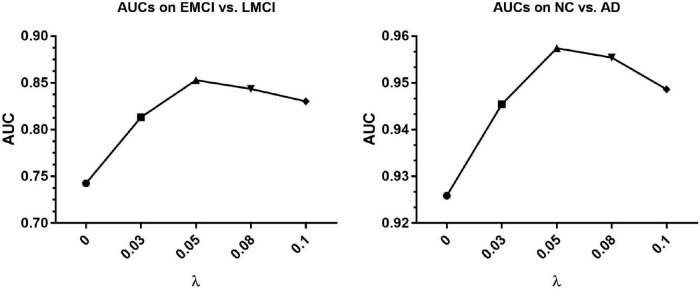
The AUCs of ablation experiments loss functions for EMCI vs. LMCI classification and NC vs. AD classification based on the Schaefer et al. (2018) atlas. AUC, area under the receiver operating characteristic curve.

To select the proper coefficient of loss functions, we compare several numbers of coefficient λ in Equation 8, including 0, 0.03, 0.05, 0.08, and 0.1. We conduct the ablation experiments based on methods in section “Experiments on Different Atlases” and the corresponding AUCs are shown in Figure 4. It can be seen that the AUC turns out to be the highest when coefficient is around 0.05 and keep at a relatively high level in the range from 0.05 to 0.08. Therefore we set coefficient λ as 0.05 in most experiments.

### Comparisons With Previous Researches

In general, there are three major advances between the proposed method and previous methods. Firstly, current PET-based methods are deficient in extracting representation features among different brain regions, incurring poor performance for the classification of early AD. The proposed BMNet introduces a bilinear pooling module into the model to explore the inter-region representation features and get a good classification performance. Secondly, there are few methods to study hard samples to improve the classification results in the brain disorder analysis. By comparison, we apply two metric learning losses to our model which has been proved useful for hard samples classification and they both get a good performance in the experiments. Thirdly, brain metabolism is very important for AD diagnosis and can only be obtained by PET images. Based on PET images, the proposed method could extract region-based features which represent the brain metabolic connectivity network, excavate the potential of PET images, and improve the diagnosis performance. This is the main superiority of inter-region-based methods with PET images compare with other modalities. In addition, the proposed PET-based method is comparable to other modalities in classification tasks between EMCI and LMCI.

### Potential Applications in Other Modalities

Considering the good performance based on FDG-PET images, the proposed BMNet including the bilinear pooling module and the metric learning loss functions also has the potential capability of diagnosis for other neurological diseases with other kinds of brain images. Besides, the proposed method only requires features of each brain region as the input. This lightweight characteristic allows the model to be easily applied to fMR and DTI images. We will try to explore more applications of the proposed method in future work.

### Limitations and Future Works

While the proposed BMNet achieves good results for the diagnosis of early AD, there are still some limitations. Firstly, considering the characteristics of the convolution neural network, the models and results are hard to be interpreted and the inter-region representation of the brain regions is hard to be visualized. Secondly, the proposed method focus on region-based features, which are lightweight but only utilize the metabolism of brain regions, limiting the ability of the network. In future work, we will try to integrate whole 3D PET images into the network to achieve joint feature extraction and classification. Finally, there is still some potential in exploiting methods that can extract brain inter-region representation features based on FDG-PET images. In the future, we will try to design methods that could extract inter-region representation features more effectively. In addition, the proposed method directly combined the contrastive loss and triplet loss with the entropy loss to better distinguish the hard samples. In the future, we will some novel designs of these losses based on domain knowledge.

### Conclusion

We propose a novel neural network method for the diagnosis of early AD with FDG-PET. We firstly construct a shallow neural network as the Baseline model. Then we introduce a bilinear pooling module into the network to try to extract inter-region representation features among the whole brain. We also apply the deep metric learning losses into the final loss function to help distinguish hard samples in the embedding space. Finally, we conduct the BMNet on the ADNI database and the results show that our method yields comparable classification performance with several representative methods. Especially, we get a satisfying classification performance in the experiment between EMCI and LMCI, which is the state-of-the-art result with FDG-PET.

## Data Availability Statement

The original contributions presented in the study are included in the article/supplementary material, further inquiries can be directed to the corresponding author/s.

## Ethics Statement

Written informed consent was obtained from the individual(s) for the publication of any potentially identifiable images or data included in this article.

## Author Contributions

WC contributed to idea conceptualization, experiments, and wrote the first draft of the manuscript. CY and CL collected data and organized the database. YP contributed to idea conceptualization. YL contributed to experiments. SC contributed to fund support and manuscript revising. XJ and JZ contributed to manuscript revising and idea conceptualization. ZY contributed to manuscript revising. XY contributed to fund support. WC, YP, JZ, XJ, and CY contributed to conception and design of the study. WC and YL performed the statistical analysis. XJ, SC, JZ, XY, and ZY wrote sections of the manuscript. All authors contributed to manuscript revision, read, and approved the submitted version.

## Conflict of Interest

The authors declare that the research was conducted in the absence of any commercial or financial relationships that could be construed as a potential conflict of interest.

## Publisher’s Note

All claims expressed in this article are solely those of the authors and do not necessarily represent those of their affiliated organizations, or those of the publisher, the editors and the reviewers. Any product that may be evaluated in this article, or claim that may be made by its manufacturer, is not guaranteed or endorsed by the publisher.
